# A Case-Study of a Child with Reversible Posterior Leukoencephalopathy Syndrome (RPLS) Associated with Severe Burns throughout the Body

**DOI:** 10.18502/ijph.v49i7.3600

**Published:** 2020-07

**Authors:** Koji WAKE, George IMATAKA, Toshihiko OHNISHI, Jin KIKUCHI, Eisei HOSHIYAMA, Kazuyuki ONO, Shigemi YOSHIHARA

**Affiliations:** 1. Department of Emergency and Critical Care Medicine, Dokkyo Medical University, Tochigi, Japan; 2. Department of Pediatrics, Dokkyo Medical University, Tochigi, Japan

## Dear Editor-in-Chief

Reversible posterior leukoencephalopathy syndrome (RPLS) was first reported by Hinchey et al in 1996 (1). In recent years, the same pathological condition has been called posterior reversible encephalopathy syndrome (PRES) (2). RPLS/PRES is characterized by the development of acute encephalopathy associated with hypertension due to various factors. Clinical symptoms include headache, vomiting, disturbance of consciousness, convulsions, cortical- and half-blindness, as well as visual impairment such as visual space neglect. Results obtained from brain magnetic resonance imaging (MRI)/ computed tomography (CT) scans of RPLS/PRES showed abnormal lesions in the occipital and parietal lobe areas (2). These symptoms are often after treatment of the underlying diseases (2). Here in this paper, we report the case of a child who developed RPLS/PRES during the treatment for severe burns throughout his body.

**Fig. 1:**
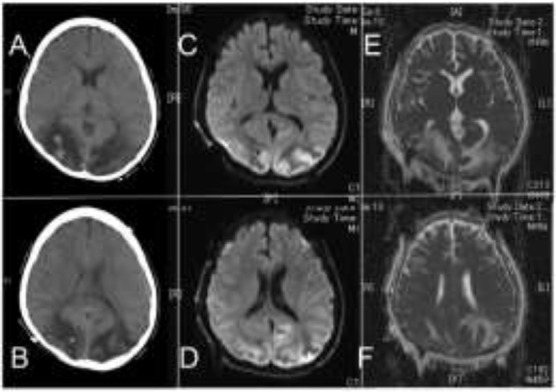
MRI findings on 54^th^ day from the onset

A healthy 13-year-old boy who suffered severe body burns from a fire at his home, was transferred to the emergency center of our hospital unit. According to the skin color tone, depth, and rule of nines, his 45% of his burns classified as deep grade III, and 30% were grade II deep skin burns. The calculated burn index was 60. Because of a burn in his airway, we immediately started ventilator treatment under general anesthesia. After 18 days of treatment in ICU, the patient advanced from artificial to spontaneous respiration. In the meantime, we used a sufficient amount of sedatives and analgesics, and performed daily skin disinfection. He also received several skin graft transplants during this period. On day 50, the patient underwent a fifth skin graft under general anesthesia. On the 51 ^st^ day, he complained about visual impairment, indicating a form of blindness. He visited an ophthalmologist, and there were no abnormalities in the light reflection, fundus, or anterior segment of the eyes. His consciousness level was E4V5M6 according to GCS scoring. His body temperature was 37.7°C, with a respiratory rate of 22 times/min. His heart rate was 86 times/min and had high blood pressure (150/89 mmHg). We determined that there was a psychiatric element and watched the course with sedatives. Patient suffered from convulsions on day 53, and a blood test showed an increase in sodium (139 mEq/L) and potassium (2.3 mEq/L), along with an overall increase in the inflammatory response. Head CT was then performed, and a low-density area with regions of high-density was found inside the bilateral occipital lobe (Fig. 1A and B). On day 54, MRI confirmed high intensity on diffusion weighted images (DWI) (Fig. 1C and D) and ADC-mapping (Fig. 1E and F) on both occipital sides.

**Fig. 2:**
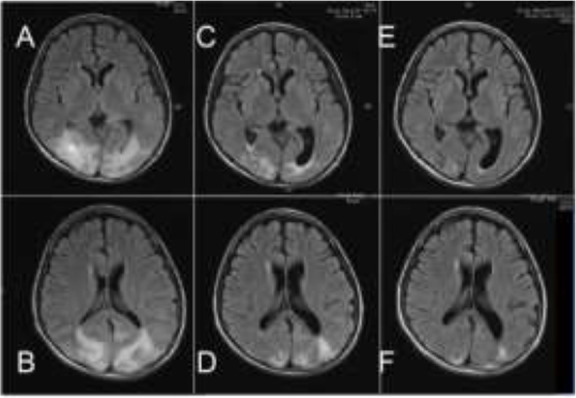
Serial MRI studies on day 61(A/B), 75(C/D) and 113(E/F)

We diagnosed reversible posterior leukoencephalopathy syndrome (PRES) based on his clinical course. The convulsions were well controlled with propofol and midazolam. Hypertension was relieved with administration of nicardipine. On day 61, MRI revealed abnormal intensity in both occipital regions with T2 weighted image and FLAIR (Fig. 2A and B). His visual impairment was recovered completely on day 64. By the day 75, a brain MRI was performed and results showed a decline in the intensity of abnormalities (Fig. 2C and D). Abnormal intensity improved further on 113^th^ day (Fig. 2E and F). The patient was discharged without motor paralysis and sequelae.

There are two major hypotheses for the development of RPLS/PRES. One is the “breakthrough” hypothesis, in which angiogenic edema is caused by the breakdown of the blood brain barrier associated with an increase in blood pressure. Alternatively, the “Vasospasm” hypothesis proposes that RPLS/PRES is caused by cerebral vasospasm. Recent studies suggested that the pathogenesis of RPLS/PRES was associated with these two mechanisms. In addition, brain MRI scans suggest that RPLS/PRES is a pathological condition in which vasogenic edema and/or cytotoxic edema is reversible. In our case, we considered that hypertensive encephalopathy was caused by several factors, including an increase in blood pressure, blood vessel dilation and angioedema caused by fluctuations in hemodynamics during burn treatment, as well as stress during the perioperative period of skin grafting. As hyper-tension is known to be a risk factor for RPLS/PRES (3). We believe that, based on the clinical case presented here, an increase in blood pressure associated with severe burns should be considered as an important factor that has the potential to trigger RPLS/PRES.
